# Fungal Endophyte *Alternaria tenuissima* Can Affect Growth and Selenium Accumulation in Its Hyperaccumulator Host *Astragalus bisulcatus*

**DOI:** 10.3389/fpls.2018.01213

**Published:** 2018-08-20

**Authors:** Stormy D. Lindblom, Ami L. Wangeline, Jose R. Valdez Barillas, Berthal Devilbiss, Sirine C. Fakra, Elizabeth A. H. Pilon-Smits

**Affiliations:** ^1^Department of Biology, Colorado State University, Fort Collins, CO, United States; ^2^Department of Biology, Laramie County Community College, Cheyenne, WY, United States; ^3^Department of Sciences and Mathematics, Texas A&M University-San Antonio, San Antonio, TX, United States; ^4^Advanced Light Source, Lawrence Berkeley National Laboratory, Berkeley, CA, United States

**Keywords:** hyperaccumulation, selenium, endophyte, *Alternaria*, *Astragalus*, x-ray analysis

## Abstract

Endophytes can enhance plant stress tolerance by promoting growth and affecting elemental accumulation, which may be useful in phytoremediation. In earlier studies, up to 35% elemental selenium (Se^0^) was found in Se hyperaccumulator *Astragalus bisulcatus*. Since Se^0^ can be produced by microbes, the plant Se^0^ was hypothesized to be microbe-derived. Here we characterize a fungal endophyte of *A. bisulcatus* named A2. It is common in seeds from natural seleniferous habitat containing 1,000–10,000 mg kg^-1^ Se. We identified A2 as *Alternaria tenuissima* via 18S rRNA sequence analysis and morphological characterization. X-ray microprobe analysis of *A. bisulcatus* seeds that did or did not harbor *Alternaria*, showed that both contained >90% organic seleno-compounds with C-Se-C configuration, likely methylselenocysteine and glutamyl-methylselenocysteine. The seed Se was concentrated in the embryo, not the seed coat. X-ray microprobe analysis of A2 in pure culture showed the fungus produced Se^0^ when supplied with selenite, but accumulated mainly organic C-Se-C compounds when supplied with selenate. A2 was completely resistant to selenate up to 300 mg L^-1^, moderately resistant to selenite (50% inhibition at ∼50 mg Se L^-1^), but relatively sensitive to methylselenocysteine and to Se extracted from *A. bisulcatus* (50% inhibition at 25 mg Se L^-1^). Four-week old *A. bisulcatus* seedlings derived from surface-sterilized seeds containing endophytic *Alternaria* were up to threefold larger than seeds obtained from seeds not showing evidence of fungal colonization. When supplied with Se, the *Alternaria*-colonized seedlings had lower shoot Se and sulfur levels than seedlings from uncolonized seeds. In conclusion, *A. tenuissima* may contribute to the Se^0^ observed earlier in *A. bisulcatus*, and affect host growth and Se accumulation. A2 is sensitive to the Se levels found in its host’s tissues, but may avoid Se toxicity by occupying low-Se areas (seed coat, apoplast) and converting plant Se to non-toxic Se^0^. These findings illustrate the potential for hyperaccumulator endophytes to affect plant properties relevant for phytoremediation. Facultative endophytes may also be applicable in bioremediation and biofortification, owing to their capacity to turn toxic inorganic forms of Se into non-toxic or even beneficial, organic forms with anticarcinogenic properties.

## Introduction

Selenium is not only toxic at elevated concentrations but also an essential micronutrient for many organisms including humans. The gap between Se deficiency and toxicity is narrow, and both are problems worldwide. Selenium is toxic due to its similarity to sulfur (S). Selenium readily replaces S in proteins, interfering with their function (Stadtman, 1990). In the Western United States, where many soils have elevated Se concentrations, chronic ingestion of high-Se plants by livestock has been reported to result in large livestock losses (Rosenfeld and Beath, 1964; Wilber, 1980).

Selenium serves no known essential function in plants, nor in fungi (Zhang and Gladyshev, 2009). In some microbes and fungi, Se is potentially used as a weak electron acceptor under anaerobic conditions (Heider and Böck, 1993). Selenium can also be beneficial to plants: it has been reported to increase growth and antioxidant activity (Hartikainen, 2005). At higher levels, Se offers plants protection against a wide variety of herbivores (Hanson et al., 2003; Freeman et al., 2006a).

Plants readily take up and assimilate Se into organic compounds, due to the similarities of Se and S (Schiavon and Pilon-Smits, 2017b). Hyperaccumulators can accumulate and tolerate up to 15,000 mg Se kg^-1^, and are also unique in that they preferentially take up Se over S and allocate Se to the reproductive tissues, i.e., flowers and seeds (Quinn et al., 2011a; Valdez Barillas et al., 2012; El Mehdawi et al., 2018). Selenium accumulation in plants can be used for phytoremediation as well as biofortification (Schiavon and Pilon-Smits, 2017a).

Several hypotheses have been proposed for why plants hyperaccumulate toxic elements like Se: inadvertent uptake, drought tolerance, elemental tolerance, allelopathy, and elemental defense against herbivores and pathogens (Boyd and Martens, 1992). For Se hyperaccumulators, the evidence for the elemental defense hypothesis is well supported. Selenium has been shown to protect plants from a variety of generalist, Se-sensitive herbivores, for a review see El Mehdawi and Pilon-Smits (2012). There is also evidence that hyperaccumulators may deposit Se in the surrounding soil as a form of elemental allelopathy against Se-sensitive neighboring plants (El Mehdawi et al., 2011a).

While Se-sensitive ecological partners suffer in their interactions with Se hyperaccumulators, Se-resistant partners may exploit the high-Se niche offered by hyperaccumulator plants. Se-resistant herbivores have been found to feed on hyperaccumulator seeds and leaves. In some of these herbivores resistance is based on tolerance and in others it is based on exclusion (Freeman et al., 2006a, 2010; Valdez Barillas et al., 2012). Furthermore, Se-tolerant neighboring plants of hyperaccumulators in the field were shown to benefit from their proximity to hyperaccumulators: they exhibited enhanced Se levels, which made them less susceptible to herbivory (El Mehdawi et al., 2011b). Selenium tolerance in these ecological partners was often associated with the accumulation of organic Se (e.g., methylselenocysteine, MeSeCys) in their tissues. Selenium hyperaccumulators also contain mostly MeSeCys, which may explain their extreme Se tolerance. MeSeCys cannot be incorporated into protein, and thus Se toxicity is avoided (Terry et al., 2000).

Relatively little is known about how Se affects the plant–microbe interactions of hyperaccumulators. Depending on whether the associated microbe lives in the rhizoplane (surface of roots), phyllosphere (surface of leaves), or as endophyte (inside plant tissues), it may experience different Se levels, and with that, Se toxicity (Valdez Barillas et al., 2011, 2012). The microbe’s relationship with the plant may involve pathogenicity, mutualism, and commensalism. Some microbes may perform beneficial functions for the hyperaccumulator: stimulating growth, aiding in nutrient and water acquisition, or fighting off pathogens. In hyperaccumulators, microbes may also affect the acquisition, speciation, and accumulation of the hyperaccumulated element (de Souza et al., 1999; Di Gregorio et al., 2006; Alford et al., 2010).

There is evidence that Se can protect plants from Se-sensitive microbial pathogens. In a study with non-hyperaccumulator *Brassica juncea*, Se was shown to protect plants from two Se-sensitive fungal pathogens, *Alternaria brassicicola* and a *Fusarium oxysporum* (Hanson et al., 2003). There is also evidence for the presence of Se-resistant microbes that live in association with hyperaccumulators (Wangeline et al., 2011). A litter decomposition experiment on seleniferous soil revealed that there were more culturable microbes (colony forming units per gram) on high-Se leaf litter from hyperaccumulators than on low-Se litter from related species collected from the same site (Quinn et al., 2011b). This finding may suggest that specialist Se-resistant decomposing microbes are present at seleniferous sites. Furthermore, a Se-resistant Rhizobacterium apparently lives in association with the hyperaccumulator *Astragalus bisulcatus* (Fabaceae), since this species produces high-Se nodules (Valdez Barillas et al., 2012). This bacterium may affect plant Se speciation, since the nodules accumulated a high fraction of elemental Se (Se^0^) (Valdez Barillas et al., 2012; Alford et al., 2014). Other endophytic bacteria were found to colonize this and other hyperaccumulators, which were also found to produce elemental Se (Staicu et al., 2015; Sura-de Jong et al., 2015).

Interestingly, roots of Se hyperaccumulators collected from the field contained high fractions of Se^0^ (up to 35%) while greenhouse-grown counterparts contained exclusively organic selenocompounds with a C-Se-C configuration (Se attached to two organic groups, Lindblom et al., 2013a). Based on these findings it was hypothesized that microbes are responsible for the production of Se^0^ observed in hyperaccumulators in their natural habitat. To test this hypothesis, hyperaccumulator plants were grown from surface-sterilized seeds and inoculated with several root-associated fungi shown earlier to be able to produce Se^0^. However, no significant effect on plant Se speciation was observed (Lindblom et al., 2013b, 2014).

In this study we test another hyperaccumulator-associated fungus that appears to be a seed-transmitted endophyte that asymptomatically colonizes stems and leaves of *A. bisulcatus* in their natural habitat. It was found to emerge regularly from surface-sterilized seeds of *A. bisulcatus*, and small spored *Alternaria* species could readily be cultured from surface-sterilized stem and leaf tissue (Valdez Barillas et al., 2012). This endophytic fungus clearly has a close association with the hyperaccumulator and thus maximal opportunity to impact plant Se speciation. In this work we identify this fungal endophyte using a combination of molecular and morphological characters, characterize the Se-related properties of the pure isolate (Se tolerance, Se metabolic properties), and test its impact on plant Se speciation, Se accumulation, and growth.

## Materials and Methods

### Biological Material

*Astragalus bisulcatus* seeds were collected in Pineridge Natural Area, Fort Collins, CO, United States during 2008–2011. The seeds were stored in coin envelopes inside a silica gel desiccator at 4°C until use. For endophyte isolation, the seeds were first surface-scarified using a scalpel blade and surface-sterilized with 50% bleach for 5 min, then rinsed three times with sterile water. Seeds were transferred to petri dishes with half strength water agar and were allowed to germinate at room temperature. Fungal mycelia growing from the seed were then transferred to half-strength malt extract agar (0.5 MEA, Difco, Detroit, MI, United States) via hyphal tipping. Hyphal tipping was repeated at least two times to ensure the fungal was a pure culture. The isolate was designated as A2.

### Sample Preparation for X-Ray Microprobe Analyses

*Astragalus bisulcatus* seeds were surface-sterilized as described above and germinated on 0.5 strength Murashige and Skoog (1962) basal salts agar medium containing 30 mg L^-1^ Na_2_SeO_4_. Two seeds were selected for X-ray microprobe analyses: one that showed the presence of A2 fungal mycelium and one that did not. These seeds were frozen at -80°C until analysis.

Agar plugs (0.5 cm × 0.5 cm) of A2 fungal mycelia were transferred to liquid malt extract medium containing 30 mg L^-1^ Na_2_SeO_4_ or 30 mg L^-1^ Na_2_SeO_3_. Sections of approximately 3 mm^3^ of A2 fungus mycelia were washed briefly in 1 mM sulfate to removed adsorbed Se. Each section was immediately placed inside a separate 0.5 ml centrifuge plastic tube, frozen in liquid nitrogen, and stored at -80°C.

### Determination of Fungal Se Tolerance

For the analysis of A2 fungal tolerance to different seleno-compounds, the fungus was cultivated under continuous fluorescent light at 22°C in sealed Petri dishes containing 0.5 strength MEA supplemented with Na_2_SeO_4_ or Na_2_SeO_3_ at 0, 10, 30, or 300 mg L^-1^. Fungal tolerance was also tested on different concentrations of MeSeCys (0, 10, 30, 60, 150 mg L^-1^) in 0.5 MEA as well as on extract made from the flowers of *A. bisulcatus* added at these same Se concentrations to 0.5 MEA.

### Fungal Identification

The A2 fungus was grown on V-8 juice agar with continuous light in unsealed plates. Potato carrot agar was used for slide culture conditions and comparison colony conditions with a 8 h light - 16 h dark cycle in unsealed plates. Morphological characterization was carried out as described below in the results section.

For molecular identification of the A2 fungus, DNA extraction, Polymerase chain reaction (PCR) and sequencing were done using the ITS 1 and 4 primers (White et al., 1990), following the protocol by Vincelli and Tisserat (2008).

### Analysis of Plant Growth and Se Accumulation as Influenced by Endophytic *Alternaria*

Seeds of *A. bisulcatus* were first scarified for 10 min with concentrated sulfuric acid, and then further surface-sterilized by rinsing for 20 min in 20% bleach, followed by five 10-min rinses in sterile water. Seeds were then germinated on sterile filter paper under continuous light at 23°C in a plant growth cabinet. Upon germination, seedlings were separated into those that naturally contained the endophyte and two that did not, and transferred to culture tubes containing autoclaved potting soil. Half of the seedlings in each group, (A2-associated and seeds without A2), were watered with 80 μM selenate in liquid 0.5 MS medium while the other half were given medium without Se. The culture tubes were sealed with breathable tape and opened only to add fresh medium. There were ten replicates per treatment (40 total). The experiment was terminated after 4 weeks. At that point half of the replicates in the control group had died (i.e., the group without the fungus and without Se added).

### Elemental Analysis

At harvest the plant roots were washed and then dried for 48 h at 45°C. Samples were digested in nitric acid as described by Zarcinas et al. (1987). Inductively coupled plasma atomic emission spectrometry (ICP-AES) was used to determine Se and S concentrations in the acid digest (Fassel, 1978).

### X-Ray Microprobe Analyses

**FIGURE 1:**
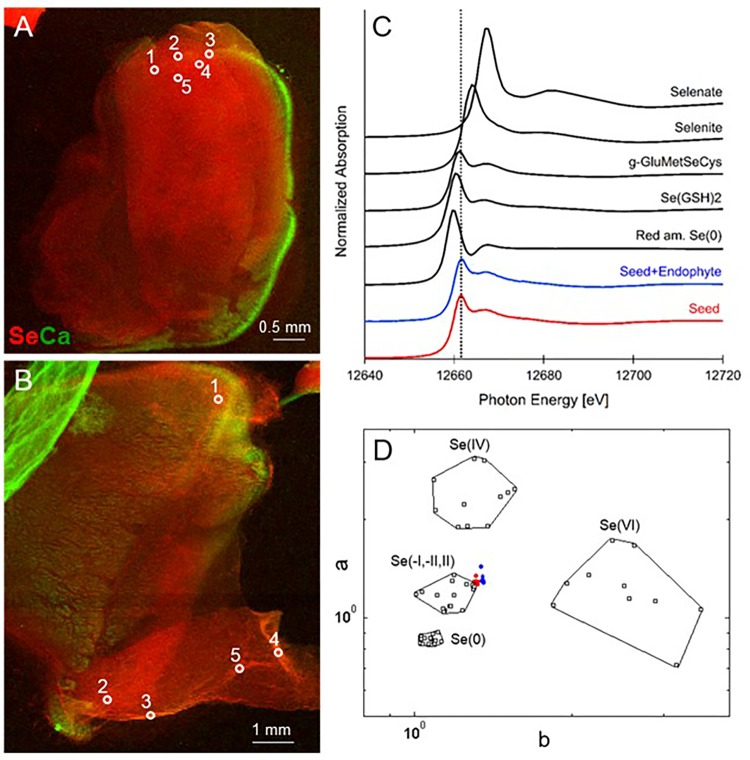
Selenium distribution and speciation in germinating *A. bisulcatus* seeds. **(A,B)** are bicolor-coded XRF maps showing the distribution of Se (in red) and Ca (in green). **(A)** Seed not containing fungal endophyte A2, **(B)** seed containing A2. Locations where XANES spectra were collected are indicated by white circles. The seedlings are oriented with their radicle at the bottom, pointing left **(A)** or right **(B)** and the cotyledons toward the top. Selenium is clearly localized in the seed embryo; the seed coat contains Ca. **(C)** Average XANES spectrum obtained from five locations for each sample, compared to Se-bearing standards. **(D)** Se valence-state scatter plot obtained from XANES spectra of the seed (red dots), seed+endophyte (blue dots) compared to Se standards (black empty squares). The hexagonal datapoints correspond to the average spectrum for each sample.

Elemental distribution and chemical speciation in the tissues were determined using μ X-ray fluorescence mapping (XRF) and μ X-ray absorption near-edge structure (XANES) spectroscopy, respectively, at the Advanced Light Source beamline 10.3.2 of the Lawrence Berkeley National Lab (Marcus et al., 2004). Frozen samples were transferred onto a Peltier stage kept at -25°C to reduce potential beam radiation damage. μXRF elemental maps were recorded at 13 keV, using a 15 μm (H) × 6 μm (V) beam, 15 μm × 15 μm pixel size, 50 ms dwell time per pixel. The chemical forms of Se in particular areas of interest were further investigated using Se K-edge XANES, at the tissue locations indicated in **Figures 1**, **4**. XANES provides information about the oxidation state and, when compared to well-characterized Se standard compounds, information about its chemical speciation (Pickering et al., 1999). XRF maps and XANES spectra were recorded with a seven element Ge solid state detector (Canberra, ON, Canada). Spectra were deadtime corrected, pre-edge background subtracted, and post-edge normalized using standard procedures (Kelly et al., 2008). Red amorphous elemental selenium (white line position set at 12660 eV) was used to calibrate the spectra. Least square linear combination (LSQ) fitting of Se XANES spectra was performed in the 12630–12850 eV range, using a library of standard seleno-compounds. As Se standards a library of 52 compounds was used (Fakra et al., 2018). All data processing and analyses were performed with a suite of custom LabVIEW (National Instruments, Austin, TX, United States) programs available at the beamline. Se valence-state scatter plots of the sample and standard compounds data were also obtained using MATLAB, following methods described in details elsewhere (Fakra et al., 2018).

### Statistical Analysis

The software JMP-IN (3.2.6, SAS Institute, Cary, NC) was used for statistical data analysis. Analysis of variance followed by a *post hoc* Tukey Kramer test was used when comparing averages of Se content and averages of plant biomass among selenium treated and untreated *A. bisulcatus* replicates. A student *t*-test was used for pairwise comparisons between two means (using an alpha error = 0.5). It was verified that the assumptions underlying these tests (normal distribution, equal variance) were met.

## Results

### X-Ray Microprobe Analysis of Seeds

When seeds of *A. bisulcatus* were surface-sterilized and germinated on sterile filter paper, about half of them contained an endophytic fungus, which was designated A2. Germination trials typically have shown around 50% infestation by *Alternaria* in *A. bisulcatus* germinated seeds based on visual estimation. To characterize the distribution and chemical speciation of Se in *A. bisulcatus* seeds, XRF and XANES analysis were performed on non-colonized vs. *Alternaria*-colonized seeds. Also, a valence plot was made, for a quick comparison of the fungal Se data with Se standards of known valence. Regardless of the presence or absence of fungus, Se was found in the embryo but not detected in the seed coat (**Figure 1**). There was no clear difference in Se speciation between *Alternaria*-colonized and uncolonized seeds (**Table 1**). Both contained predominantly (86–90%) organic Se with C-Se-C configuration, that fitted best with the Se standard γ-glutamyl-methylSeCys but may also include other C-Se-C compounds like SeMet or methyl-SeCys. In both seeds, there were small fractions of other selenocompounds that correspond with Se(IV) and Se(VI) oxidation states (forms of selenite and selenate, respectively) that fitted best with various metal selenate standards (Zn, Fe, and Cu selenate, particularly). The micro-XRF spectra (MCA) that were collected on each Se XANES spot indeed detected Ca, Fe, Zn, and Cu; at the energy we were exciting the sample with (13 keV), we were not very sensitive to elements below Ca (such as K, Cl, and S).

**Table 1:** *Astragalus bisulcatus* seed Se speciation results obtained from least squares linear combination fitting (LCF) of the XANES spectra collected at the spots shown (as white circles) in **Figure 1**, using 52 standard seleno-compounds.

Seed	NSS (xE-4)	C-Se-C (%)	Fe-Se(IV) (%)	Zn selenate (%)
**No endophyte**				
Spot 1	3.9	92	4	2
Spot 2	3.8	90	5.5	1.5
Spot 3	6.1	89.2	3	5
Spot 4	3.7	95.3	1.4	1.3
Spot 5	4.1	93.4	1.9	2.5
Mean		92.0	3.2	2.5
SEM		1.1	0.7	0.7
**With endophyte**				
Spot 1	7.7	76	12.6	9
Spot 2	1.8	90	3.3	5.2
Spot 3	6.4	87.7	3.6	5.5
Spot 4	6.25	87	4	6
Spot 5	5.6	93	2	3
Mean		86.7	5.1	5.7
SEM		2.9	1.9	1.0


*The best LCF was obtained by minimizing the normalized sumsquares residuals [NSS = 100 × Σ(μ_*exp*_ – μ_*fit*_)^2^/Σ(μ_*exp*_)^2^], where μ is the normalized absorbance. Error on percentages is estimated to be +10%. Replicates represent individual spectra obtained from different locations in the seeds. SEM: standard error of the mean. The Fe-Se(IV) corresponds to the standard mandarinoite.*

**FIGURE 2:**
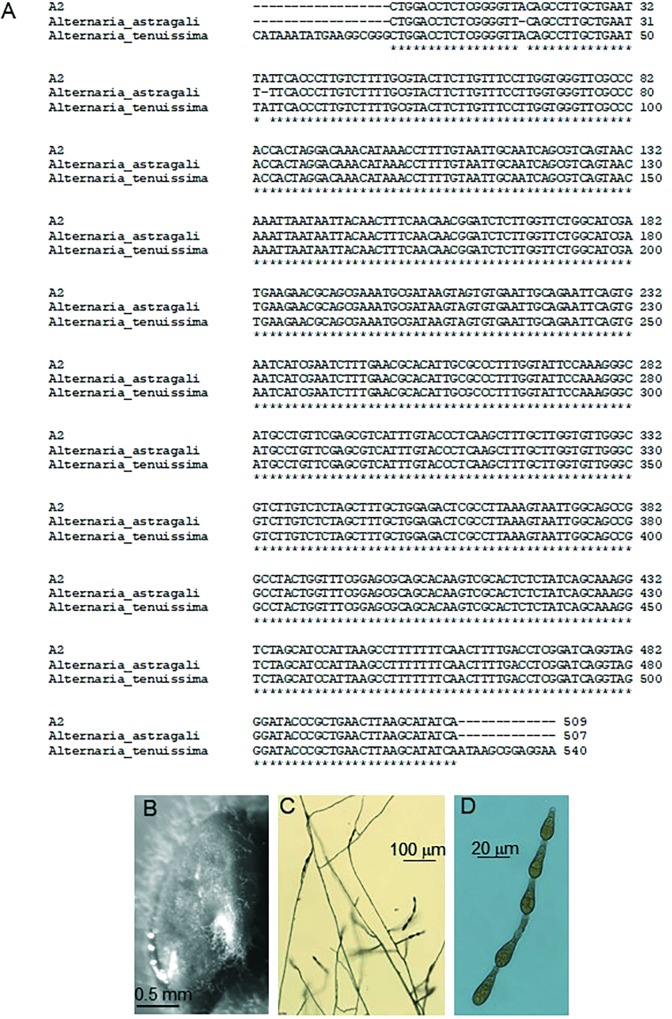
Information used to identify A2 fungus. **(A)** ITS sequence alignment with *Alternaria astragali* (99%) and *Alternaria tenuissima* (100%). **(B)** Surface-sterilized *A. bisulcatus* seed with A2 endophyte mycelia emerging from the seed coat. **(C)** Conidiophores and conidia of A2, and **(D)** magnified conidia.

The identity of A2 was initially investigated by DNA sequencing of the internal transcribed spacer region of ribosomal genes (ITS 1 and 4) of the small ribosomal subunit. As shown in **Figure 2A**, the sequence from A2 showed 100% sequence similarity with the known plant pathogen *Alternaria tenuissima* and 99.6% similarity with *Alternaria astragali*, a rhizosphere fungus associated with *A. bisulcatus* (Wangeline and Reeves, 2007). Small-spored *Alternaria*, particularly *A. alternata* and *A. tenuissima*, are difficult to distinguish using solely molecular techniques (Andrew et al., 2009), so morphological characteristics were included for identification (Simmons, 2007). The references were updated accordingly (Simmons, 2007). To date *A. tenuissima* is grouped among other small-spored *Alternaria* that show no association between host, geographic origin and phylogenetic lineage, and is considered by some as an unresolved group associated with *Alternaria alternata* (Andrew et al., 2009).

### Fungal Isolate A2 Taxonomic Description

The A2 fungal strain was identified as *A. tenuissima* (Nees and T. Nees: Fr.) Wiltshire, anamorph (no known teleomorph), with the following specific morphology.

Colony at 7–8 days, PCA and V-8: colony 5–6 cm diameter. On PCA is a surface network of interwoven radial hyphae, gray to buff with little sporulation and no discernible rings. Also present is a dense mat of reduced aerial hyphae. The reverse is dark green to black and uniform. The colony on V-8 is similar to PCA. Mycelium cottony, with an advancing colony edge of approximately 10 mm. Colony has dark mycelial rings alternating with dense aerial rings when infrequently present. Mycelia gray, buff, and medium to dark brown. Mycelia layers upward with increasing age. The reverse is dark green to black.

Conidiophores (40×) produced on V-8 range on average between 130–155 μm in length (**Figures 2C,D**). Juvenile conidia are ovoid and have no definable beak, while most mature into a body narrowly ellipsoid with a long beak. The conidiophores are mostly simple but may have low amounts of branching (1–2), sometimes proliferating at 1–2 conidiogenous sites. Conidia chains of 2–6 (8) are produced (most common 4). Older areas of mycelial growth can have a distinctive rope-like formation, commonly up to or less commonly surpassing 11 μm wide.

Conidium bodies (100×) are ovoid or long ellipsoid; commonly with a long beak or less commonly with no distinctive beak. Conidium length, with beak, ranging from 14 to 39 μm with 2–5 transverse septa, width ranging from 6 to 11 μm with 0–3 longitudinal septa, and beak length from 1.5 to 17 μm. Noteworthy is that mature conidia have beaks >10 μm, and continue to divide, becoming septate in both the beak (2–3 cells) and body gaining additional longitudinal septa. Also noteworthy that roughly 50% of conidia have zero longitudinal septa. Few conidium walls are rough or thickened.

### Selenium Tolerance of the *A. tenuissima* A2 Strain

**FIGURE 3:**
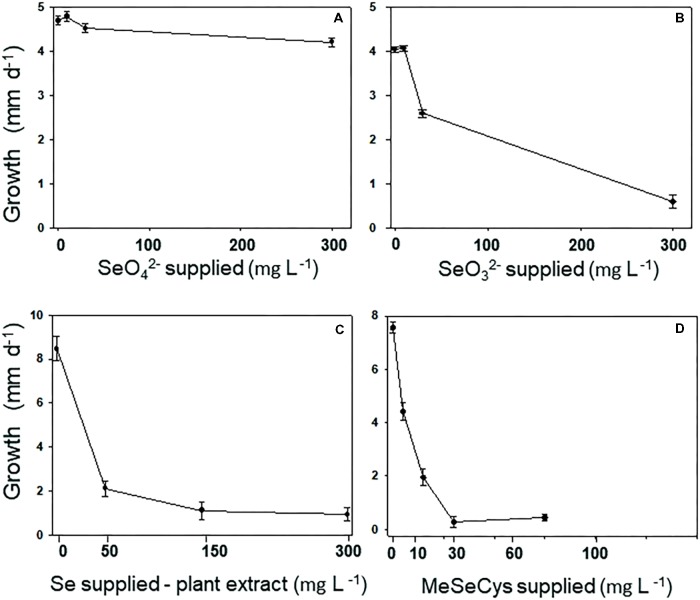
Fungus A2 is resistant to selenate, moderately resistant to selenite, but relatively sensitive to organic MeSeCys and hyperaccumulator-derived Se (= MeSeCys). A2 resistance was measured as growth of the colony per day on varying concentrations of **(A)** selenate, **(B)** selenite, **(C)** extract from the flowers of *Astragalus bisulcatus*, and **(D)** methyl-selenocysteine.

The Se tolerance of the A2 fungus to different forms and levels of Se when grown on MEA is shown in **Figure 3**. The fungus was most tolerant to selenate: its growth was still 90% of the control when supplied with 300 mg Se L^-1^ (**Figure 3A**). The fungus was also fairly tolerant to selenite: it showed 50% inhibition around 100 mg Se L^-1^ (**Figure 3B**). To test A2 growth as a function of plant-derived Se, flower material was extracted in water and added to the growth medium at different dilutions. The Se concentration in the extract was determined using ICP-AES. The fungus was significantly inhibited by the plant extract, showing 50% inhibition around 25 mg Se L^-1^ (**Figure 3C**). To test whether this was likely due to the Se, particularly C-Se-C, or (also) to other growth inhibiting compounds in the flowers, fungal growth was also determined as a function of MeSeCys concentration. When pure MeSeCys was added to the medium, the growth of A2 was 50% inhibited around 20 mg Se L^-1^ (**Figure 3D**), i.e., A2 growth was similarly inhibited by pure MeSeCys and by the Se extracted from *A. bisulcatus*.

### X-Ray Microprobe Analysis of A2 Mycelium Grown on Medium With Selenate or Selenite

**FIGURE 4:**
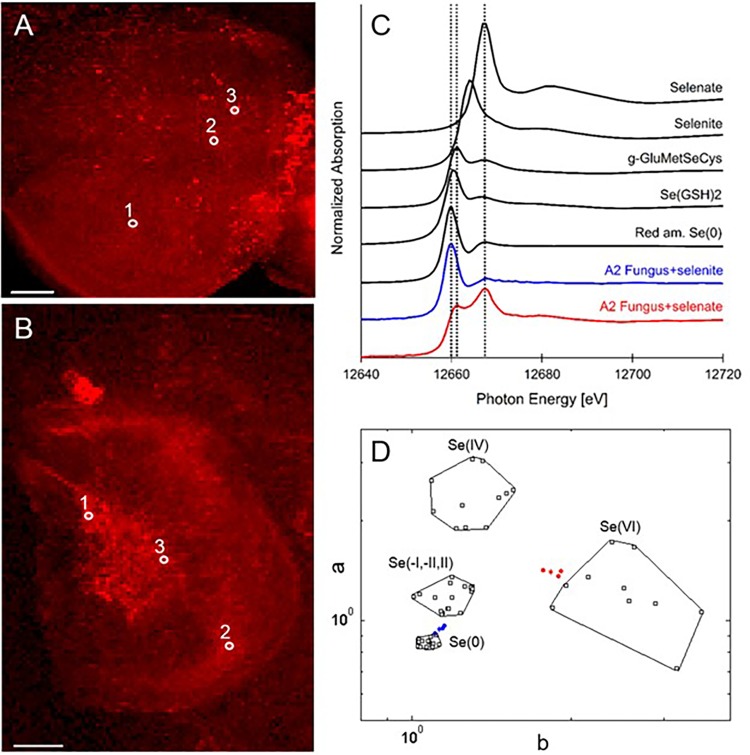
Selenium distribution and speciation in A2 fungal material (mycelium) collected after growth on MEA medium spiked with selenate or selenite. **(A)** XRF Se map of A2 supplied with selenate. **(B)** XRF Se map of A2 supplied with selenite. Scale bars are 500 microns. **(C)** Average XANES spectrum obtained from three locations for each sample (white circles in **A,B**), compared to Se standards. **(D)** Se valence-state scatter plot obtained from XANES spectra of A2 supplied with selenate (in red), A2 supplied with selenite (in blue), compared to Se standards (in black). The hexagonal datapoints correspond to the average spectrum for each sample.

X-ray microprobe analysis was carried out on mycelia of A2 that was grown on fungal growth media spiked with selenate or selenite. XRF maps and XANES spectra are shown in **Figure 4**, as well as a valence plot for a quick comparison of the fungal Se data with Se standards of known valence. The detailed Se speciation results are listed in **Table 2**. When the fungus was supplied with selenite (SeO_3_^2-^), 83% of Se in the mycelia was present as Se^0^; the remainder was best matching the seleno-diglutathione (Se-GSH_2_) standard. When supplied with selenate (SeO_4_^2-^) the fungus accumulated a large fraction (67%) of Se as C-Se-C, as well as a substantial fraction of selenate (27%), and a minor fraction of Se-GSH_2_ (6%).

**Table 2:** Selenium speciation results obtained from least squares LCF of experimental XANES spectra collected from the locations shown in **Figure 4** with standard seleno-compounds.

A	NSS (xE-4)	C-Se-C (%)	MeSeCys (%)	Se(GSH)2 (%)	Mg/Cu selenate (%)	SeCl4 (%)
**A2 selenate**						
Spot 1	2.2	68	0	7	29	0
Spot 2	5.7	60	0	11	31	0
Spot 3	7.0	74	74	0	22	7
Average		67	25	6	27	2
**B^∗^**	**NSS (xE-4)**	**Red Se(0) (%)**	**Black Se(0) (%)**	**Gray Se(0) (%)**	**Se(0) total (%)**	**Se(GSH)2 (%)**
**A2 selenite**						
Spot 1	2.3	81	0	11	92	8
Spot 2	3.0	30	45	0	75	25
Average		55	23	6	83	17


*The best LCF was obtained by minimizing the normalized sumsquares residuals [NSS = 100 × Σ(μ_*exp*_ – μ_*fit*_)^2^/Σ(μ_*exp*_)^2^], where μ is the normalized absorbance. Error on percentages is estimated to be +10%. XANES spectra were collected on *Alternaria* sp. originally isolated from *Astragalus bisulcatus* seeds, grown on malt extract agar with 30 mg L^-*1*^ sodium selenate (SeO_*4*_^*2*-^) or sodium selenite (SeO_*3*_^*2*-^). Replicates represent individual spectra obtained from mycelial mass. ^∗^Spot 3 not reported; too noisy to fit.*

### Effect of *A. tenuissima* (A2) on *A. bisulcatus* Growth and Se and S Accumulation

**FIGURE 5:**
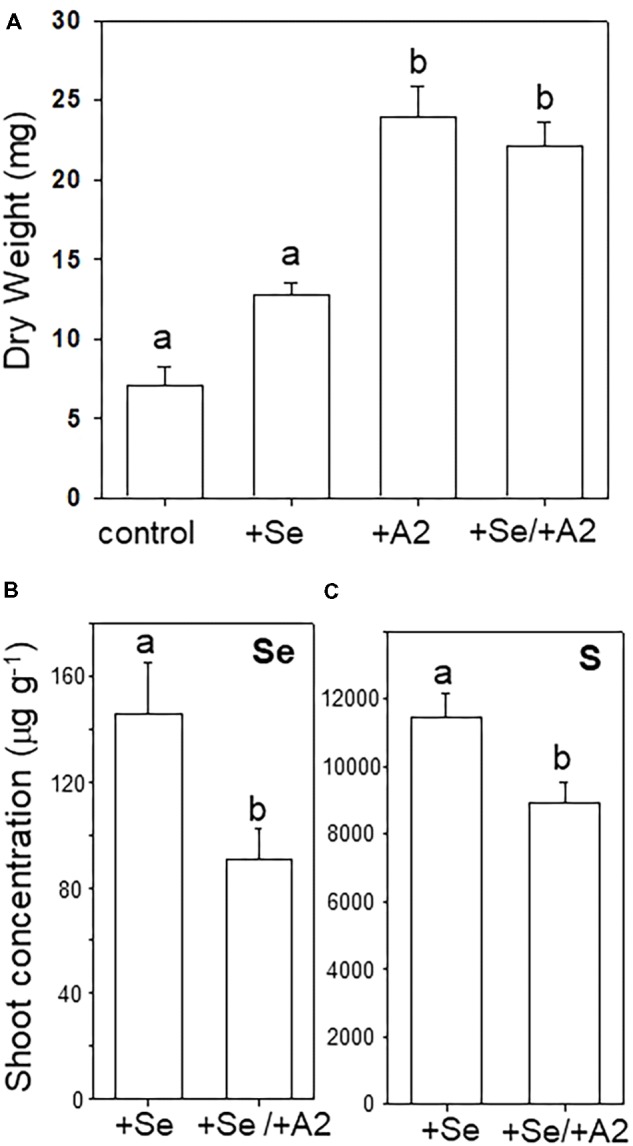
Effect of the presence of *Alternaria* endophyte on growth and Se and S accumulation in its host, Se hyperaccumulator *A. bisulcatus*. **(A)** Plant biomass. **(B)** Shoot Se concentration, **(C)** shoot sulfur concentration. Different letters above bars indicate significant differences (*p* < 0.05) between treatment means (*n* = 10).

Seeds with hyphae emerging during germination were separated from those that did not show hyphae, and the two groups were cultivated on sterile peat moss for 4 weeks with or without selenate, the main form of bioavailable Se found in soil. The A2-containing seedlings had reached a twofold to threefold greater dry weight compared to uncolonized seedlings (**Figures 5A,B**). The addition of Se did not significantly affect *A. bisulcatus* growth for either group (**Figures 5A,B**). The presence of the A2 fungus was also associated with reduced shoot Se and S levels (**Figures 5C,D**).

## Discussion

Hyperaccumulators of toxic elements are an interesting potential resource for discovery of microbes with properties useful in phytoremediation or bioremediation. Like all plants, Se hyperaccumulators harbor a variety of endophytic and rhizosphere bacteria and fungi (Wangeline et al., 2011; Sura-de Jong et al., 2015). While Se hyperaccumulator *A. bisulcatus* accumulates Se mainly in organic C-Se-C forms (methyl-SeCys, especially), up to 30% elemental Se^0^ was reported in the roots and stem of mature field-collected plants, as well as in seedlings germinated from field-collected seeds (Valdez Barillas et al., 2012; Lindblom et al., 2013a). The A2 fungal strain, identified here as *A. tenuissima*, may contribute to this fraction of Se^0^ in *A. bisulcatus*. It could be cultured from about 50% of field-collected, surface-sterilized *A. bisulcatus* seeds. In addition, small-spored *Alternaria* species, of which *A. tenuissima* is a member, could be cultured from surface-sterilized roots and stems of *A. bisulcatus*. In pure culture supplied with selenite, the A2 fungus was shown here to be capable of producing Se^0^. While *A. tenuissima* is known as a potential plant pathogen with a wide host range (Mishra and Parakash, 1975), there is no evidence from this study that it acts as a pathogen on *A. bisulcatus*. *Alternaria*-containing seedlings grew better than seedlings not containing this endophyte, so it actually may be growth-promoting. However, it is possible that the relationship between *A. bisulcatus* and A2 depends on the conditions, particularly the Se level, the overall nutrient supply and the health status of the plant.

The Se speciation in germinating *Alternaria*-colonized or uncolonized *A. bisulcatus* seeds was similar: 86–90% C-Se-C. This was likely MeSeCys and glutamyl-MeSeCys, as has been previously reported for seeds (Nigam and McConnell, 1969); MeSeCys has also been found to be the main form of Se in leaves (Freeman et al., 2006b) and flowers of this species (Valdez Barillas et al., 2012). In contrast, an earlier study by Valdez Barillas et al. (2012) found a seed in a late-stage *Alternaria* infestation to contain 22% Se^0^, both in the seed and the mycelium growing from the seed. These results suggest that this *Alternaria* can convert the C-Se-C in the seed to Se^0^, perhaps as a tolerance mechanism. Grown in pure culture, A2 produced Se^0^ when supplied with selenite. Conversion of more toxic forms of Se to insoluble, inert Se^0^ is known to be a tolerance mechanism for many microbes (Gharieb et al., 1994 and citations therein; Gadd, 1993; Lovely, 1993). A2 appears to have a different Se resistance mechanism for selenate, since it contained a variety of organic selenocompounds (C-Se-C) when supplied with this form of Se. The tolerance of A2 to selenate was much higher than for selenite. Surprisingly, although A2 grew very well on seeds that contained upward of 1,000 mg Se kg^-1^ (Galeas et al., 2007; Quinn et al., 2011a), it was already 50% inhibited by 25 mg kg^-1^ Se extracted from *A. bisulcatus* flowers, as well as by 20 mg kg^-1^ MeSeCys. A possible explanation for the ability of A2 to successfully grow on these high-Se plants could be that A2 occupies areas of the plant where there is relatively less Se, such as the interface between the seed coat and the seed embryo, and in the apoplast. As shown here, the seed coat contains very little Se, and in earlier studies energy dispersive X-ray analysis of hyperaccumulator leaves revealed that Se is generally stored in the vacuole in Se hyperaccumulators, and not in the apoplast (Freeman et al., 2006c, 2010). Thus, A2 may not encounter toxic Se levels in the living plant, like it does when grown on pure selenocompounds or homogenized plant extract.

The ITS sequence alignment identification of the fungus revealed an interesting similarity to another fungal-symbiont of *A. bisulcatus*, *A. astragali* (A3), which was originally isolated from the rhizoplane of surface-sterilized roots (Wangeline and Reeves, 2007). An additional *Alternaria* species, *A. seleniiphila* (A1) was isolated from the rhizoplane of hyperaccumulator *Stanleya pinnata* (Wangeline and Reeves, 2007). Both A1 and A3 were characterized for Se tolerance and speciation by Lindblom et al. (2013a). The Se-related characteristics of A2 are somewhat similar to A1 and A3. All are capable of reducing selenite to Se^0^ and all are fairly tolerant to selenate. All three also stimulated the growth of their hyperaccumulator host.

Perhaps related to its effect on Se speciation toward more insoluble Se^0^ in roots, the A2-containing *A. bisulcatus* seedlings showed significantly lower Se and S levels in their shoots. In previous studies where hyperaccumulators were inoculated with the related *Alternaria* species A1 and A3, there was a reduction in root-to-shoot translocation (Lindblom et al., 2013b, 2014). The same may be the case for A2; the root biomass was too small to determine root elemental concentrations. The possible mechanism for reduced translocation could be the production of Se^0^ in the rhizosphere or inside the root apoplast, trapping Se in a non-soluble and therefore non-translocatable form. In this context it is interesting to note that pure A2 cultures produced mainly C-Se-C compounds from selenate (the form provided in the seedling study), and elemental Se from selenite. Thus, if the A2 endophyte produced elemental Se in the root, the plant may have reduced the selenate to selenite first.

This study helps us understand the ecology of *Alternaria* fungi in relation to various hosts. This fungus is best known for its capacity to colonize different hosts including many domesticated crop species, where it may or may not act as a pathogen. Zou et al. (2018), however, report an *Alternaria* sp. fungal endophyte that acted as a plant growth promoting fungus. The increases in shoot and root biomass observed in that study were attributed to a plant metabolic upregulation induced by the fungal endophyte. In this non-domesticated Se hyperaccumulator *A. tenuissima* behaves asymptomatically similarly to other vertically transmitted fungal endophytes, perhaps due to the host’s elemental defense (the high levels of this toxic element may negatively affect pathogens) and limited access to nutrients by the host’s apoplast. However, it is apparently capable of colonizing the hyperaccumulator’s tissues and perpetuating its genetic line by keeping its host alive and colonizing the seeds. This is another example of *Alternaria’s* phenotypic plasticity, showing its ability to colonize a diverse range of hosts via different mechanisms and under different types of ecological interactions.

## Author Contributions

SL performed the experiments and wrote the manuscript. AW and BD helped with the fungal identification and advised concerning fungal cultivation. SL, SF, and JVB helped with X-ray microprobe analysis. EP-S oversaw the project and helped with manuscript preparation.

## Conflict of Interest Statement

The authors declare that the research was conducted in the absence of any commercial or financial relationships that could be construed as a potential conflict of interest.
